# Supplementary material for: Upstream Regulator Analysis of Wooden Breast Myopathy Proteomics in Commercial Broilers and Comparison to Feed Efficiency Proteomics in Pedigree Male Broilers
Source: Foods. 2021 Jan 6;10(1):104. doi: 10.3390/foods10010104 (PMC7825620; doi:10.3390/foods10010104)
Supplement: Supplementary file 1 [file foods-10-00104-s001.pdf]

Supplementary Table S1.

Abbreviations and names with P value and fold change of proteins presented in Tables 2 and 3 listed alphabetically. Fold Differences in green (negative values) were down-regulated in wooden breast (WB) myopathy whereas fold differences in red (positive values) were up-regulated in WB myopathy breast muscle relative to values expressed in normal breast muscle tissue.

Symbol	Entrez Gene Name	P-value	Fold Change
ABCB6	ATP binding cassette subfamily B member 6	0.003	-1.78
ACP1	acid phosphatase 1	0.031	-1.20
AK1	adenylate kinase 1	0.005	-1.76
ALDOC	aldolase, fructose-bisphosphate C	0.009	-1.43
ANXA2	annexin A2	0.017	1.95
ARF1	ADP ribosylation factor 1	0.015	1.46
C3	complement C3	0.016	1.75
CALR	Calreticulin	0.014	1.60
CAPNS1	calpain small subunit 1	0.034	-1.20
CBR1	carbonyl reductase 1	0.027	-1.33
CDH13	cadherin 13	0.024	1.54
COL7A1	collagen type VII alpha 1 chain	0.002	-1.29
CRYAB	crystallin alpha B	0.049	1.72
CS	citrate synthase	0.048	-1.20
CTSB	cathepsin B	0.051	1.82
DDX3X	DEAD-box helicase 3 X-linked	0.006	1.31
DES	Desmin	0.005	16.10
DSTN	destrin, actin depolymerizing factor	0.001	1.80
DYNC1H1	dynein cytoplasmic 1 heavy chain 1	0.019	1.33
EEF1A1	eukaryotic translation elongation factor 1 alpha 1	0.000	2.68
EIF3L	eukaryotic translation initiation factor 3 subunit L	0.042	1.50
FDPS	farnesyl diphosphate synthase	0.032	-1.53
FGA	fibrinogen alpha chain	0.008	1.45
FLNA	filamin A	0.041	2.12
FSCN1	fascin actin-bundling protein 1	0.047	2.67
GAPDH	glyceraldehyde-3-phosphate dehydrogenase	0.033	-1.47
GATD3A/GATD3B	glutamine amidotransferase like class 1 domain containing 3A	0.001	-1.84
GLO1	glyoxalase I	0.032	-1.61
GOT1	glutamic-oxaloacetic transaminase 1	0.004	-1.49
GOT2	glutamic-oxaloacetic transaminase 2	0.019	-1.37
GPD1	glycerol-3-phosphate dehydrogenase 1	0.039	-1.73
HINT1	histidine triad nucleotide binding protein 1	0.017	-2.14
HK1	hexokinase 1	0.028	1.59
HNRNPH1	heterogeneous nuclear ribonucleoprotein H1	0.045	1.25
HSP90AA1	heat shock protein 90 alpha family class A member 1	0.000	1.18

HSP90B1	heat shock protein 90 beta family member 1	0.003	1.55
HSPA2	heat shock protein family A (Hsp70) member 2	0.035	1.31
HSPA5	heat shock protein family A (Hsp70) member 5	0.002	1.38
IARS1	isoleucyl-tRNA synthetase 1	0.039	1.21
IGHM	immunoglobulin heavy constant mu	0.028	1.83
KLHL40	kelch like family member 40	0.017	2.96
LDHA	lactate dehydrogenase A	0.006	-1.96
ME1	malic enzyme 1	0.028	-1.56
P4HB	prolyl 4-hydroxylase subunit beta	0.006	1.72
PARK7	Parkinsonism associated deglycase	0.016	-1.74
PDCD6IP	programmed cell death 6 interacting protein	0.020	1.32
PDIA3	protein disulfide isomerase family A member 3	0.003	1.52
PDIA4	protein disulfide isomerase family A member 4	0.007	1.83
PDIA6	protein disulfide isomerase family A member 6	0.005	1.30
PEBP1	phosphatidylethanolamine binding protein 1	0.041	-1.46
PGAM1	phosphoglycerate mutase 1	0.022	-1.98
PGD	phosphogluconate dehydrogenase	0.014	1.40
PGK1	phosphoglycerate kinase 1	0.026	-1.70
PKM	pyruvate kinase M1/2	0.016	-1.93
PLS3	plastin 3	0.011	2.73
POSTN	Periostin	0.024	2.36
PPIA	peptidylprolyl isomerase A	0.031	1.52
PRDX6	peroxiredoxin 6	0.044	-1.28
RAB1A	RAB1A, member RAS oncogene family	0.001	1.44
RACK1	receptor for activated C kinase 1	0.015	2.54
RAN	RAN, member RAS oncogene family	0.049	1.20
RHOA	ras homolog family member A	0.005	1.67
RPL11	ribosomal protein L11	0.010	3.44
RPL18	ribosomal protein L18	0.033	2.49
RPL6	ribosomal protein L6	0.011	3.25
RPLP0	ribosomal protein lateral stalk subunit P0	0.027	2.58
RPS11	ribosomal protein S11	0.024	1.83
RPS14	ribosomal protein S14	0.006	2.37
RPS2	ribosomal protein S2	0.007	2.54
RPS24	ribosomal protein S24	0.025	1.85
RPS3	ribosomal protein S3	0.010	3.46
Rps3a1	ribosomal protein S3A1	0.013	2.91
RPS7	ribosomal protein S7	0.022	1.86
RPS8	ribosomal protein S8	0.009	2.33
TAGLN2	transgelin 2	0.033	1.52
TLN1	talin 1	0.013	2.85
UBE2V2	ubiquitin conjugating enzyme E2 V2	0.005	-1.22
VIM	vimentin	0.010	3.41

YWHAE	tyrosine 3-monooxygenase/tryptophan 5-monooxygenase activation protein epsilon	0.010	1.66
YWHAZ	tyrosine 3-monooxygenase/tryptophan 5-monooxygenase activation protein zeta	0.010	1.53

Supplementary Table S2

Functions predicted to be inhibited (blue) in breast muscle of Pedigree Male Broilers exhibiting a high compared to low feed efficiency phenotype (previously unpublished data from Kong et al. [11]).

Functions	Activation *z*-Score	*p*-Value of Overlap	Differentially Expressed Proteins [1]
Necrosis of Muscle	−2.78	3.33×10^{-7}	ACTC1, ATP2B4, CAMK2D, CAV1, DMD, EEF1A1, EEF1A2, GPX1, KLHL40, NUB1, PRDX3, PSMB1, SGCG
Cell death	−2.60	1.54×10^{-6}	ACTC1, ATP2B4 CAMK2D CAV1, DMD, EEF1A1, EEF1A2, GPX1, KLHL40, NUB1, PRDX3 PSMB1 SOD1
Apoptosis	−2.81	1.29×10^{-6}	ACTC1, ATP2B4, GPX1, KLHL40, NUB1, PRDX3 PSMB1 SOD1
